# Comparative study on optic disc features of premature infants and full‐term newborns

**DOI:** 10.1186/s12886-021-01833-6

**Published:** 2021-03-06

**Authors:** Xiaofen Feng, Yan Nan, Jiandong Pan, Ruitao Zou, Lijun Shen, Feng Chen

**Affiliations:** 1 School of Ophthalmology & Optometry, Eye Hospital, Wenzhou Medical University, Wenzhou, Zhejiang China; 2 Ruian Maternity and Child Care Hospital, Wenzhou, Zhejiang China

**Keywords:** Premature infant, Full‐term newborns, Optic disc parameter, Shape of sclerotic ring and optic disc, Wide-Field Digital Pediatric Retinal Imaging System

## Abstract

**Background:**

To study optic disc features of premature infants and compare to that of term infants to explore the pattern and features of newborn optic disc development and provide the basis for the diagnosis of newborn optic disc disease.

**Methods:**

This was a prospective clinical research. Newborns underwent newborn fundus disease screening from January 1st, 2016 to October 31st, 2016 in the neonatal ward of Ruian City Maternal and Child Health Hospital were selected. RetCam 3 Version6.1.25.0 Wide-Field Digital Pediatric Retinal Imaging System developed by Clarity Medical Systems, Inc was adopted to conduct fundus examination on both eyes, 130 degree wide-angle lens was used to film the images centering optic disc.

**Results:**

For both premature infants and full-term newborns, vertical diameter of the optic disc to lateral diameter of the optic disc ratio was > 1, and the shape of the optic disc was a vertical oval. The difference of each optic disc parameter between premature infants and full-term newborns was not statistically significant (*P* > 0.05). There’s a difference of constitution of sclerotic ring type on optic disc between premature infants and full-term newborns. Among which, the proportion of single ring type and double ring type in premature infants was higher than that in full-term newborns (*P* < 0.05). The proportion of no ring type in full-term newborns was higher than that in premature infants (*P* < 0.05). The proportion of mixed type had no significant difference (*P* > 0.05) between premature infants and full-term newborns.

**Conclusions:**

We found that The proportion of mature types (single ring type and double ring type) in full-term newborns was higher than that in premature infants. While there’s no statistical difference of the proportion of mixed types between premature infants and full-term newborns. Double ring type was a normal stage of the development of optic disc.

## Background

The optic disc, also called the optic nerve head, was a reddish disc-shaped structure with a diameter of about 1.5 mm, about 3 mm from the macula a lutea to the nasal side. The optic disc was the part of the retina where the optic fibers collect through the eye and the beginning of the optic nerve. The optic disc had no light sensation due to the lack of photoreceptor cell, therefore, forming a physiological blind spot in the visual field. When seeing normally, the blind spots in one eye’s field of view could be compensated by the visual field of the contralateral eye, so no blind spot would be felt in the field of vision.

The optic disc developed from the embryo’s stalk. All the retinal ganglion cell axons and retinal blood vessels needed to pass through the optic disc. Thus even minor optic disc injury might cause serious clinical consequences[1]. Any pathological influence of ganglion axon in anatomical path from the retina to the brain could cause characteristic visual field defects, regulate optic disc loss and paleness. These characteristic changed contributed to clinical location of the lesion. Therefore, studies of the morphology of optic discs had always been the focus of researchers. Understanding its development was of great significance to the diagnosis and treatment of certain diseases such as optic nerve disease, retinopathy, glaucoma. Neonatal eye diseases had their characteristics in anatomy, physiology, pathology, clinical manifestations, diagnosis, treatment methods and other aspects. The fundus data measured from normal adults was not suitable criterion for juvenile fundus screening. Therefore, it was necessary to establish a Chinese visual disc parameter system for newborns to provide reference and basis for the diagnosis and treatment of neonatal diseases.

Newborn group lacked capacity of communication, cooperation with inspection, as well as equipment, only some relatively simple instruments were used for measurement, such as direct or indirect ophthalmoscope. This required fairly skilled level of ophthalmic instrument operation of the physician to avoid omissions. For the recording of the optic disc, the examination results were usually recorded by pictures or description. There were differences in subjective description between different doctors, lacked of objective diagnostic evidence, that would influence early diagnosis and monitoring of diseases, which might lead to delay in diagnosis and treatment.

In recent years, with the rapid development of digital imaging systems, especially the neonatal digital wide-area fundus imaging system (Retcam) developed by Clarity Medical Systems in the United States, had been widely used in screening for neonatal eye diseases. Retcam had the characteristics of instant imaging, real and clear image, as well as could accurately record and further analyzed the image, including the degree and extent of the lesion. In addition, that made it possible to quantify numerical structure of the neonatal fundus structure according to the conversion relationship between the pixels measured by the lens provided by the manufacturer and the actual millimeter value. More studies on the morphology and parameters of optic discs in neonates, especially premature babies appeared recently. Compared with domestic studies, researches mainly focused on retinopathy of prematurity [2–4], while premature infants and full-term newborns reports on the study of pediatric optic disc morphology were very rare [5–9]. Therefore, we designed this research topic, by taking pictures of the fundus of premature and full-term newborns, observing the morphological characteristics of the scleral ring of the optic disc, then recording and converting the specific values of the optic disc parameters of premature and full-term newborns. After comparing and analyzing the optic disc characteristics of premature and full-term newborns, the specific values of optic disc parameters in premature and full-term newborns in China would be summarized, that could provide a diagnostic basis for optic disc diagnosis and treatment of optic nerve diseases as well as congenital glaucoma.

## Methods

### Research population

Select newborns screened for neonatal fundus disease in the neonatal ward of Ruian Maternal and Child Health Hospital from January 1, 2016 to October 31, 2016. Including 60 premature infants (31.74 weeks to 36.86 weeks of gestational age, 33.57 ± 1.11 weeks of average gestational age), and 60 full-term newborns (37.14 weeks to 40.85 weeks of gestational age, average gestational age 39.50 ± 1.52 weeks). Among the premature infants, 30 (50 %) males and 30 females (50 %) received neonatal fundus screening within one week of birth (corrected gestational age 32.28 weeks to 37.71 weeks, mean corrected gestational age 34.19 ± 0.70 weeks). In full-term newborns, 30 males (50 %) and 30 females (50 %) received neonatal fundus screening within 1 week (corrected gestational age 37.14 weeks to 41.71 weeks, mean corrected gestational age 39.97 ± 1.52 weeks). All the guardians of the inspected were informed and signed the informed consent form. The study was approved by the Ethics Committee of the Eye Hospital of Wenzhou Medical University.

The inclusion criteria for premature infants were (1) gestational age within 37 weeks; (2) within 1 week after birth; (3) physical condition could be matched with screening;(4) delivery or laparotomy. The inclusion criteria for full-term newborns were (1) gestational age of 37 to 40 weeks; (2) birth weight over 2500 g; (3) within 1 week after birth; (4) delivery or laparotomy. Exclusion criteria were (1) birth injury and other major systemic diseases (such as abnormal brain development, cardiopulmonary disease, respiratory failure) and could not be screened for neonatal fundus; (2) eye infections and inflammation affect neonatal fundus screening operations; (3) eye disease affects the morphology of the optic disc; (4) optic disc congenital dysplasia affects the morphology of normal optic disc.

### Retcam

Developed by Clarity Medical Systems, Retcam (Wide-Field Digital Pediatric Retinal Imaging System) V6.1.25.0 used computer image acquisition software with five lenses to collect real-time dynamics of the anterior segment of the eye (including the cornea, anterior chamber angle, iris and lens) and the fundus (including the optic nerve and retina). Retcam instruments had advantages over traditional binocular indirect ophthalmoscopy [10]. Retcam was easy and convenient to operate and complete the inspection in few minutes. Retcam also had a wide range of 130° lens and high resolution, which could accurately determine the lesions and retinal vascularization. And Retcam generally took 5 photos to complete the screening of neonatal eye disease (the posterior pole and 4 quadrants), could complete the fundus examination and diagnosis of the disease within a few minutes. In addition, photo images could be saved and exported in different formats for further analysis of the extent and extent of the lesion after the end of the examination. These images could also be used for ROP training, student teaching, and explaining the condition of the child’s parents.

### Fundus screening operation

Operation of each newborn was performed by the same experienced child ocular specialist. We used the Compound Tropicamide Eye Drops to the eyes of the newborns 40 minutes before the test, waiting for the pupil to be fully dilated. We placed the newborns on the inspection table and fixed it with a wax bag. We added Proparacaine Hydrochloride Eye Drops to the neonatal conjunctival sac for 3 minutes, and used the newborn-specific speculum to open the speculum and fully exposed the eyeball after the surface anesthesia was fully effective. Ofloxacin Eye Ointment was used to make the RetCam probe couplant, then placed the lens vertically above the eye, gently touched the corneal surface, and followed the order of the posterior pole, the top, the temporal side, the lower side, and the nasal side. Deleted unclear pictures, clear fundus photos were saved and archived.

### Data collection

We exported all the photos from the neonatal digital wide-area fundus imaging system instrument in jpg format without compression, and uniformly selected the right eye clear photos. We measured the video disc parameters used Markman v2.7.17, a professional measurement and annotation software developed by the Adobe AIR platform. All disc measurements and conversion of the optic disc were performed by the same researcher.

### Disc measurement

**Fig. 1:**
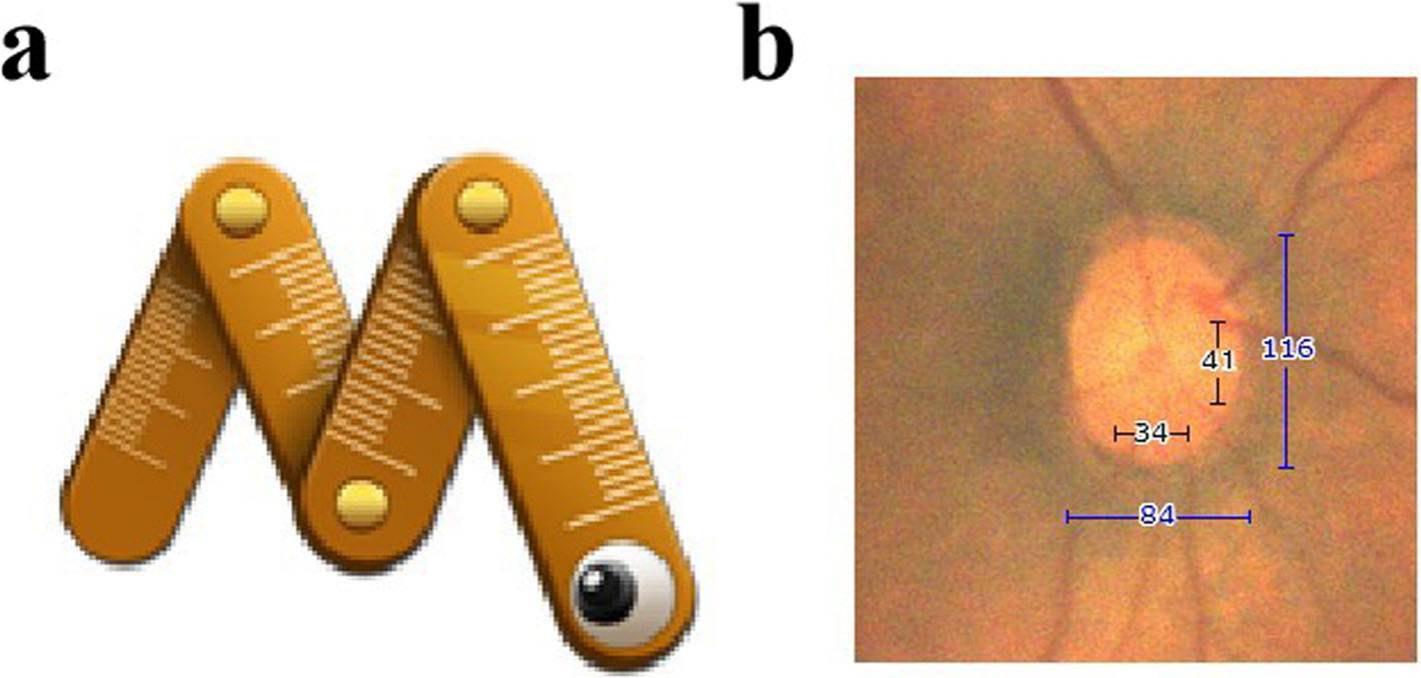
Equipment and methods. **a** MarkMan V2.7.17. **b** Measurement of the optic disc (in pixels). The image was freely available to use

The parameters of the optic disc, including the longitudinal diameter of the optic disc, the transverse diameter of the optic disc, the longitudinal diameter of the optic cup, the transverse diameter of the optic cup, the average diameter of the optic disc, the ratio of the cup to the pan and the cup to pan were measured in this study. The selected fundus photos were imported into the Markman V2.7.17 software (Fig. 1a), ignored the distortion of the camera system. Taken the edge of the optic disc as the boundary of the optic disc, according to the morphological characteristics of the neonatal disc, defined the vertical diameter of the optic disc as the longest diameter from 11 o’clock to 1 o’clock to 5 o’clock to 7 o’clock. The horizontal diameter referred to the shortest path from 8 o’clock to 10 o’clock to 2 o’clock to 4 o’clock. We used the same method to measure the vertical and horizontal diameters of the optic cup [11] (Fig. 1b).

### Unit conversion

According to the data provided by Retcam [12], it was the third generation instrument V6.1.25.0 version, with imaging pixel up to 1.92 million, of which the horizontal pixel was 1600 and the vertical pixel was 1200. And the conversion relationship between the pixel of Retcam instrument and the actual value was 0.0122mm/pixel. According to the conversion relationship between pixels and millimeters, the longitudinal and transverse diameters of the optic disc and the optic cup were converted from pixel units to millimeter units, and the average diameter of the optic disc was then calculated.

### Statistical analysis

Statistical data were analyzed with SPSS 22.0 (IBM. Armonk, NY, USA). Statistical analysis was performed on all data collected, and the optic disc morphology, optic disc scleral ring type and optic disc parameters of premature infants and full-term newborns were summarized. The chi-square test was used to compare the differences between the four different types of optic discs in premature infants and full-term newborns. An independent sample *t*-test was used to compare the differences between disc diameter and cup diameter in premature infants and full-term newborns, *P* < 0.05 (* or #) was considered statistically significant.

## Results

### Basic information of the research subjects

**Fig. 2:**
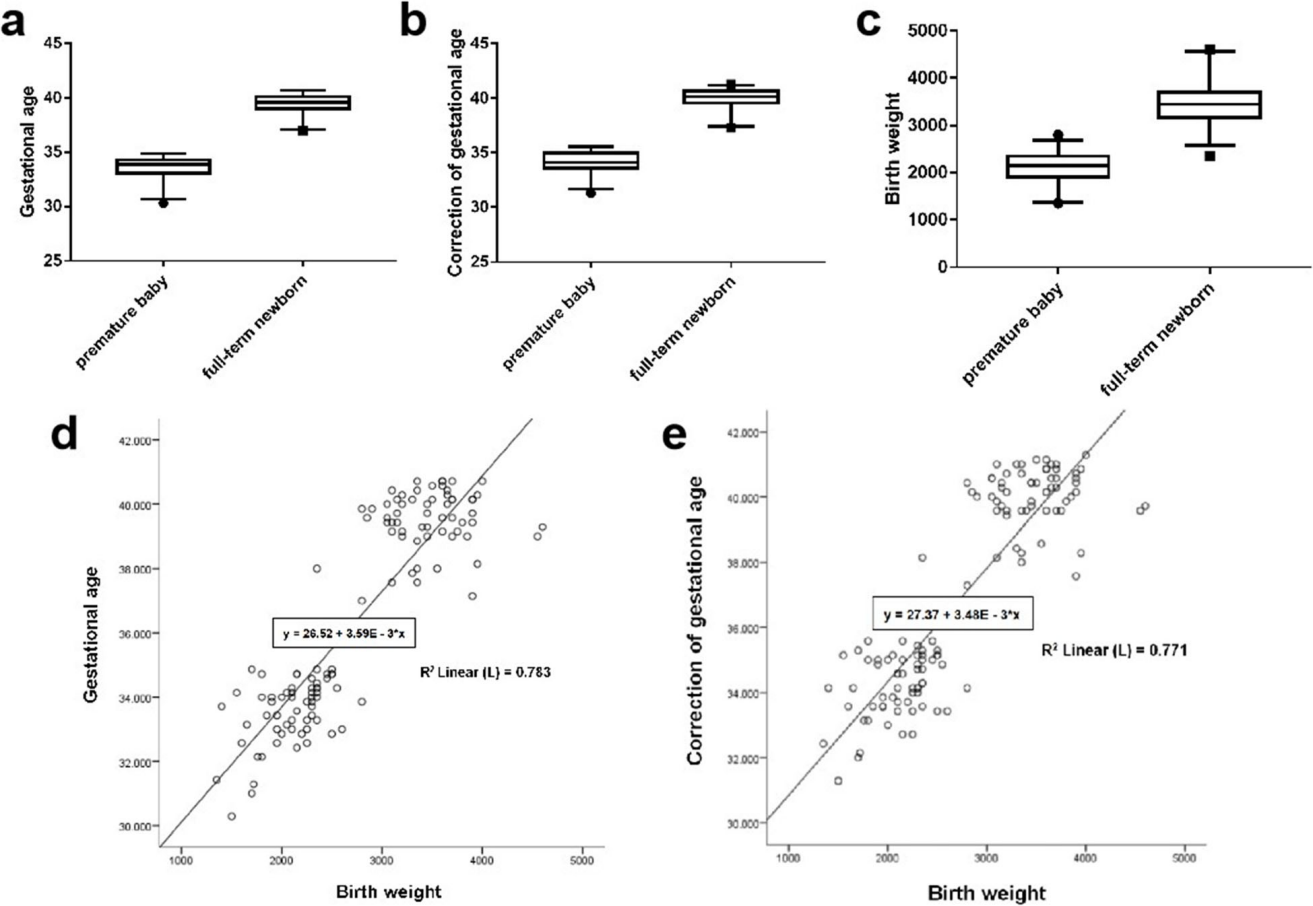
Basic information of the research object. **a** Comparison of gestational age distribution between premature and full-term neonates. **b** Comparison of corrected gestational age distribution between premature and full-term neonates. **c** Comparison of birth weight distribution between premature and full-term neonates. **d** Scatter plot of birth weight and gestational age in preterm and term newborns. **e** Scatter plot of birth weight and corrected gestational age in preterm and term newborns

120 newborns (120 eyes), including 60 premature infants (30 males and 30 females), 60 full-term newborns (30 males and 30 females) were chosen in our study. Newborns were screened for neonatal fundus within one week after birth. The average gestational age, corrected gestational age, birth weight of premature and full-term newborns were shown in Table 1. The gestational age, corrected gestational age, and specific distribution of birth weight were represented by box plots (Fig. 2a-c). The correlation between birth weight and birth gestational age as well as between birth weight and corrected gestational age were represented by scatter plots (Fig. 2d, e). The birth weight of the premature baby would influence the correct development of the optic nerve and the retina.

**Table 1:** Basic information on premature and full-term neonates

	Premature baby	Full-term newborn
Number of cases (eye)	60	60
Average birth gestational age (week)	33.57 ± 1.11	39.50 ± 1.52
Average corrected gestational age (week)	34.19 ± 0.70	39.97 ± 1.52
Average birth weight (g)	2110.5 ± 106.07	3466.68 ± 106.67

The birth weight, gestational age and corrected gestational age of premature and full-term newborns measured by the experiment were found normally distributed by nonparametric test (single sample K-S test). Result of *t* test showed that the birth weight, corrected gestational age and birth gestational age were the same in the two groups, indicating that the three indicators have the characteristics of variance and homogeneity. The birth weight was tested by *t* test. The difference between the two groups was statistically significant (*t*=-20.515, *P* = 0.000). It could be considered that the birth weight levels between the two groups were different, and the second group was significantly higher than the first group. The corrected gestational age was statistically significant between the two groups (*t*=-32.887, *P* = 0.000), indicated that the difference between the two groups was statistically significant, and the second group was significantly higher than the first group. The difference in gestational age between the two groups was statistically significant (*t*=-33.351, *P* = 0.000), indicated that the difference between the two groups was statistically significant, and the second group was significantly higher than the first group.

### Video disc parameters

**Fig. 3:**
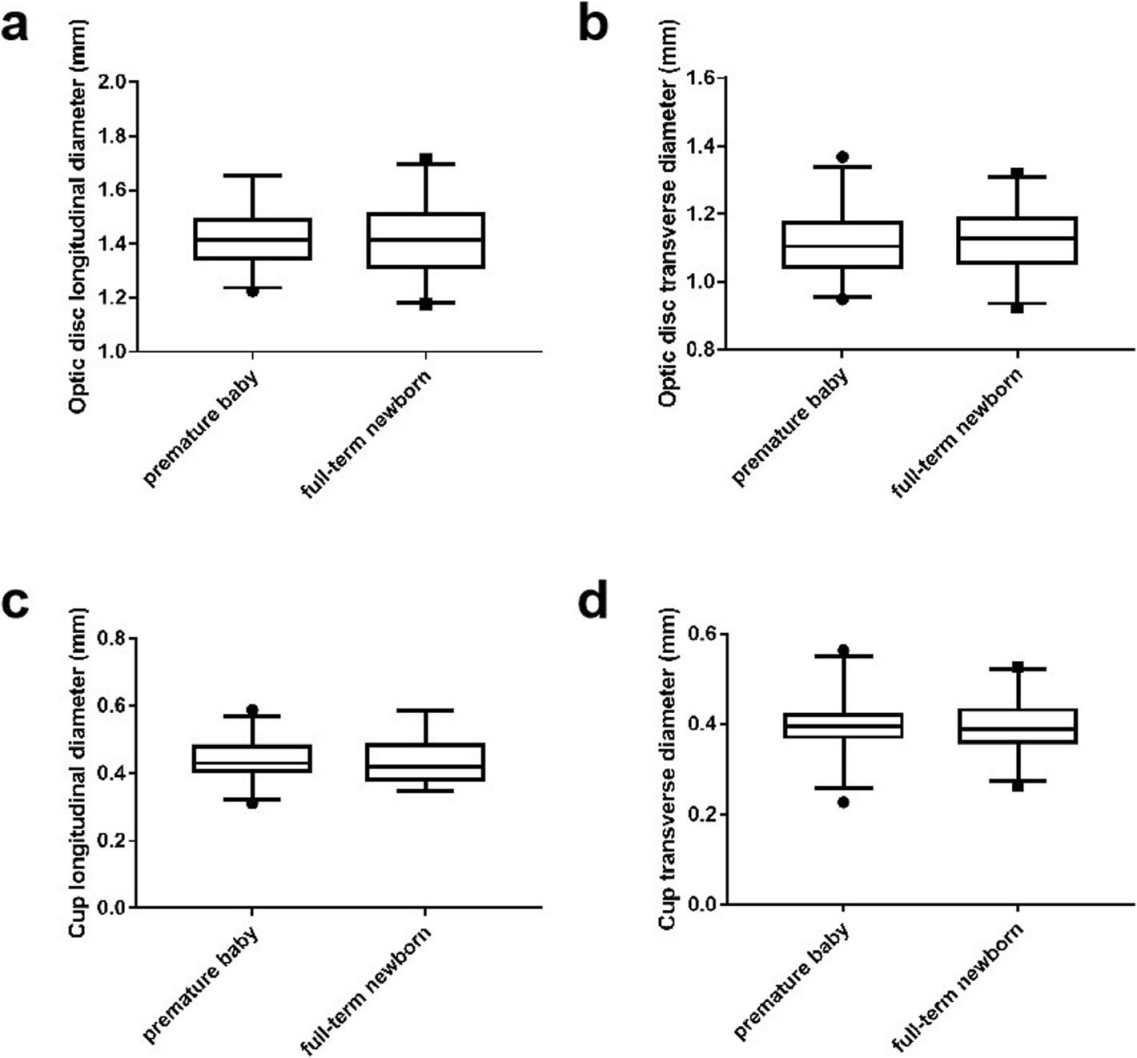
Disc parameter. **a** Comparison of longitudinal diameter distribution of optic disc in premature and full-term neonates. **b** Comparison of transverse diameter distribution of optic disc in premature and full-term neonates. **c** Comparison of longitudinal diameter distribution of premature infants and full-term neonates. **d** Comparison of transverse diameter distribution of premature infants and full-term neonates

Average disc length, optic disc transverse diameter, optic disc longitudinal diameter/optical disc transverse diameter, optic disc average diameter, optic cup longitudinal diameter, optic cup transverse diameter, cup-and-panel longitudinal ratio, cup-to-disk lateral ratio of premature and full-term newborns were shown in Table 2. The distribution of the longitudinal diameter and transverse diameter of the optic disc in premature and full-term newborns, the longitudinal diameter and the transverse diameter of the optic cup were compared using a box diagram (Fig. 3a-d). The longitudinal diameter and transverse diameter of the optic disc, as well as the longitudinal diameter and the transverse diameter of the optic cup in premature and full-term newborns were found normally distributed by two independent sample *t*-tests, the results showed that *P* > 0.05, indicated that premature and full-term newborns were no difference in optic disc parameters.

**Table 2:** Value table of specific parameters of premature and full-term neonatal optic discs. (*n* = 120)

	Premature baby	Full-term newborn
Number of cases (eye)	60	60
Disc longitudinal diameter (mm)	1.43 ± 0.00	1.42 ± 0.11
Optic disc transverse diameter(mm)	1.11 ± 0.04	1.13 ± 0.05
Vertical diameter / transverse diameter	1.29 ± 0.01	1.27 ± 0.01
Average diameter of the optic disc (mm)	1.27 ± 0.02	1.27 ± 0.08
Depending on the longitudinal diameter of the cup (mm)	0.44 ± 0.01	0.44 ± 0.03
Sight cup diameter (mm)	0.40 ± 0.01	0.40 ± 0.05
C/D (vertical)	0.31 ± 0.01	0.31 ± 0.05
C/D (horizontal)	0.36 ± 0.01	0.35 ± 0.03

### Disc shape

The average visual disc longitudinal diameter/opic disc transverse diameter of preterm infants included in this study was 1.29 ± 0.01, and the full-length neonatal disc longitudinal diameter/opic disc transverse diameter was 1.27 ± 0.01, and the ratio was higher than full-term newborns. Therefore, the optic discs of premature and full-term newborns were vertical oval discs, and no transverse elliptical discs were found in this study. In addition, the color of the optic disc was mostly pink or reddish, because some of the premature infants had residual vitreous primitive arteries, covered the surface of the optic disc, which might affect the observation of some cases of physiological depression. The optic disc borders of premature and full-term newborns were blurred compared to adult optic discs, and the borders were relatively irregular.

### Optic disc scleral ring morphology

**Fig. 4:**
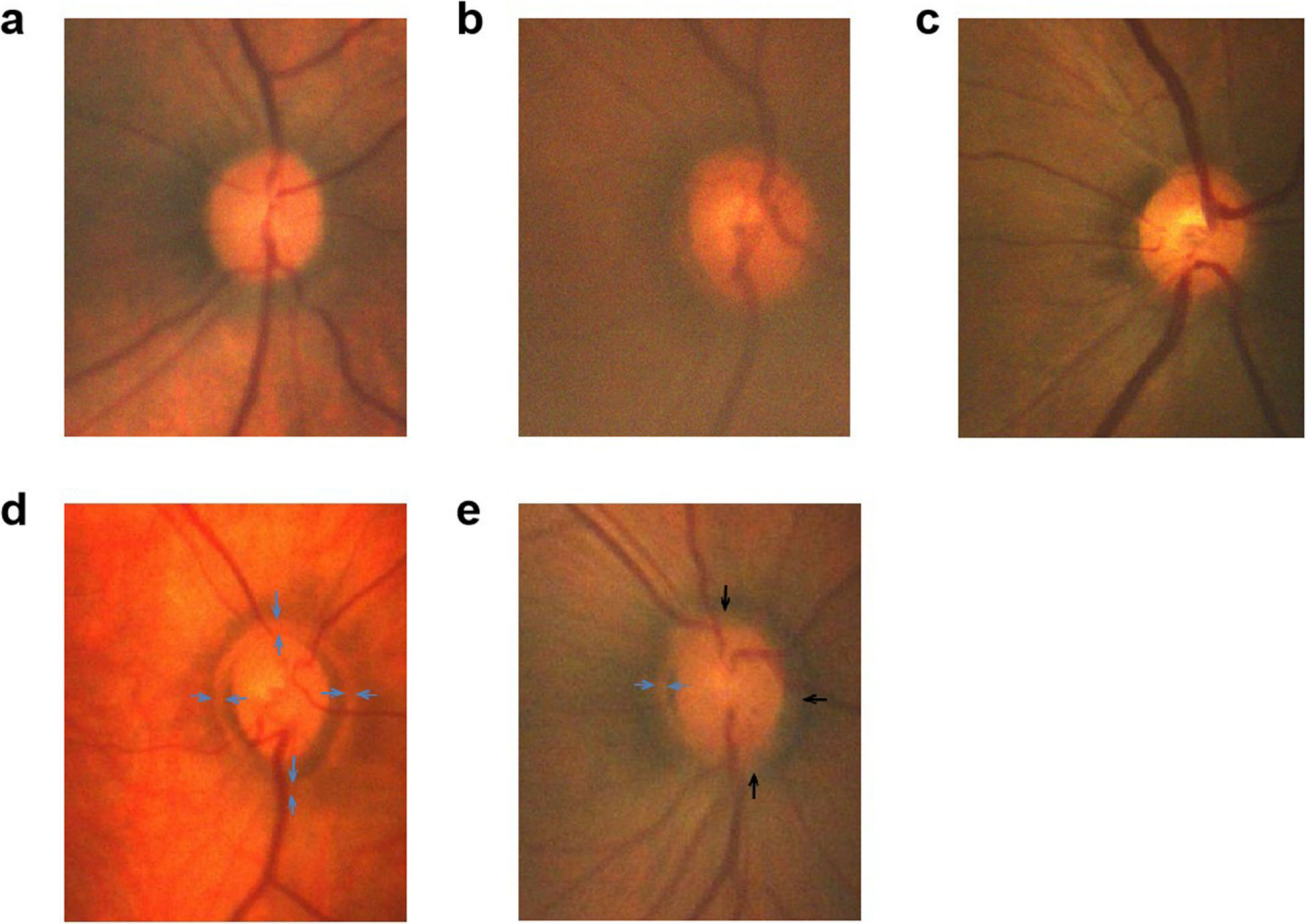
The shape of optic disc and optic disc scleral ring. **a** Optic disc scleral ring acyclic. **b** Optic disc scleral single ring. **c** Optic disc scleral ring double loop. **d** Disc sclera hybrid ring (single-double). The optic disc was surrounded by a double ring (blue arrow). **e** Disc scleral ring hybrid (single-none). Double rings were seen locally on the temporal side of the optic disc (blue arrow), and the rest were single rings (black arrow)

Most of the optic discs were surrounded by a scleral ring which presented four types, including acyclic (Fig. 4a), single ring (Fig. 4b), double ring (Fig. 4c) and mixed (Fig. 4d, e) types. Melanin filling could be observed in part of the optic disc and the scleral ring, which was especially evident in the double scleral ring. The distribution of optic disc scleral rings in preterm and term neonates was shown in Table 3.

**Table 3:** Distribution of scleral ring types in optic discs in premature and full-term neonates

	Scleral ring type of optic disc				Total
	Acyclic type (%)	Single ring type (%)	Double ring type (%)	Hybrid (%)	
Premature baby	6 (10.0)	22 (36.7)	17 (28.3)	15 (25.0)	60
Full-term newborn	28 (46.7)	11 (18.3)	4 (6.7)	17 (28.3)	60

Chi-square test showed the proportion of optic disc scleral ring morphology in preterm and full-term newborns was not the same (χ^2^ = 26.075, *P* = 0.000). Further, through the layered chi-square test, it was found that there was a significant difference between the two groups in the single-ring type of the scleral ring of the optic disc (χ^2^ = 5.058, *P* = 0.025). The scleral ring of the optic disc and the bicyclic pattern of the optic disc scleral ring also had a statistically significant difference between the two groups (χ^2^ = 19.863, *P* = 0.000; χ^2^ = 9.755, *P* = 0.002). The irregular shape of the optic disc scleral ring was not statistically significant between the two groups (χ^2^ = 0.170, *P* = 0.680). Through the composition ratio of the optic disc scleral ring in the premature infant group and the full-term newborns group, the monocyclic type of the optic disc scleral ring had a higher proportion in preterm infants, and the proportion of the double ring of the optic disc scleral ring was significantly higher in preterm infants than in full-term newborns. The acyclic form of the optic disc scleral ring was significantly higher in full-term newborns, and the irregular shape of the optic disc scleral ring weren’t significant difference between the two groups.

## Discussion

The optic disc was the beginning of the optic nerve, was traversed by the retinal ganglion axon fibers in the nerve fiber layer and converged to the optic disc following the fine structure of the retina. All retinal ganglion axons passed through the optic disc. The optic disc was consisted of the front area of the screen (between the screen and the glass body), the screen area and the rear area of the screen. The blood circulation of the optic disc was mainly from choroid around the optic disk which supplied by the Haller-Zinn arterial ring and the posterior ciliary artery. Since the structure of the optic disc included all retinal ganglion cell axons and retinal blood vessels, even relatively small optic disc damage could cause serious clinical consequences. Any case in the anatomical pathway from the retina to the brain would affects the ganglion axons, caused a characteristic visual field defect. Therefore, many researchers had paid great attention and enthusiasm to the study of the morphology of optic discs over the years.

Understanding the characteristics and developmental rules of normal optic disc was of great significance for the diagnosis and treatment of diseases such as optic nerve disease, retinopathy and glaucoma. The size and shape of the optic disc in normal healthy eyes might vary greatly in different regions, races, stages of development and age, the appearance might be extremely asymmetric. Therefore, before identified the pathological changes of the optic disc, it was necessary to grasp the morphological characteristics and developmental rules of the optic disc of the normal eye, so as to avoid missed diagnosis and misdiagnosis. The morphological characteristics of adult optic discs were significantly different from those of infants and young children, the size of optic disc and optic nerve at birth was 75 % of that of adults. Neonatal eye disease had special characteristics in anatomy, physiology, pathology, clinical manifestations, diagnosis and treatment methods, as well as other aspects [13–15].

Previous studies mostly focused on the characteristics of adult optic discs. Fundus data measured from normal adults were not suitable screening criteria for neonatal fundus screening. Therefore, to establish a visual disc parameter system for newborns was of necessity, which provided a reference and basis for the diagnosis of neonatal fundus diseases, especially for the diagnosis and treatment of optic nerve diseases and glaucoma.

Because of the lack of neonatal fundus examination equipment, previous study of neonatal fundus was to analyzed the optic disc shape of children over 10 years old, and to evaluate the relationship between optic disc parameter morphology and birth gestational age or birth weight in the past. Fledelius et al. [16] noted that children with birth weight less than 2000 g had significantly greater disc C/D than full-term newborns, but there was no significant difference between children less than 2000 g and 1500 g. Hellstrom et al. [10, 17] reported that children at the age under 7 years had an average premature birth of 27 weeks, the optic disc and disc area were significantly smaller compared with full-term newborns, and there was no difference in the C/D area between the two groups. In a study of 12-year-old children, Samarawickrama et al. [18] indicated that infants with intrauterine growth retardation had significantly greater optic disc C/D ratios than the normal group. It is suggested that intrauterine growth retardation might be a risk factor of glaucoma. Other studies also reported a significant increase in optic disc C/D ratio in infants with ischemic brain injury [17, 18]. However, these studies analyzed the disc shape of premature or full-term newborns over 7 years of age did not represent the optic disc characteristics of the neonatal period.

In our study, 60 preterm infants were at a chronological gestational age of 31.74 to 36.86 weeks, with an average gestational age of 33.57 ± 1.11 weeks, average corrected gestational age of 34.19 ± 0.70 weeks, and the average birth weight of 2110.5 ± 106.07 g. The full-term gestational age of 60 full-term newborns ranged from 37.14 weeks to 40.85 weeks. The average gestational age was 39.50 ± 1.52 weeks, the average corrected gestational age was 39.97 ± 1.52 weeks, and the average birth weight was 3646.68 ± 106.67 g. Neonatal fundus screening was performed within one week of birth. There were significant differences in gestational age, corrected gestational age, and birth weight between the preterm infants and full-term newborns. Disc parameters of premature infants showed that longitudinal diameter of the optic disc was 1.43 ± 0.00mm, the transverse diameter of the optic disc was 1.11 ± 0.04mm, the longitudinal diameter of the optic cup was 0.44 ± 0.01mm, the transverse diameter of the optic cup was 0.40 ± 0.01mm, the ratio was 0.31 ± 0.01, and the transverse cup-to-disk ratio was 0.36 ± 0.01. Full-term newborns optic disc parameters showed that longitudinal diameter of the optic disc was 1.42 ± 0.11mm, the transverse diameter of the optic disc was 1.13 ± 0.05mm, the longitudinal diameter of the optic cup was 0.44 ± 0.03mm, the transverse diameter of the optic cup was 0.40 ± 0.05mm, longitudinal the cup-to-disk ratio was 0.31 ± 0.05, and the transverse cup-to-disk ratio was 0.35 ± 0.03.

The neonatal optic disc parameters obtained in this study were compared with those of normal adults. Typical adult optic disc was elliptical with an average longitudinal diameter (1.9 mm) that was slightly larger than the average horizontal diameter (1.7 to 1.8 mm). In this study, we found that the visual disc longitudinal diameter/transverse diameter ratio was 1.29 in preterm infants and 1.27 in full-term newborns, indicated the morphology of optic disc was vertical elliptical and the number of transverse elliptical optic disc was 0. This might indicate that the visual system in the neonatal period was at the beginning of development. The shape of the optic disc was mainly larger than the transverse diameter, and was mainly vertical and elliptical. The development of the eyeball and the development of the transverse diameter of the optic disc were accelerated with age, some may exceed the longitudinal diameter and develop into a transverse elliptical optic disc.

Current study of neonatal optic discs mainly focused on optic discs in preterm infants in different premature stages of preterm infants development, including premature infants with fixed eyeballs in formalin due to premature death [1, 19], birth babies [19, 20] and premature birth children [10, 17, 20, 21]. Different methods have also been introduced into analytical research. Binoculars and other methods [11, 17, 22–24] were used in some studies and the in vivo digital images used in this study all measured higher value than those measured by Rimmmer at autopsy. This might due to use of formalin fixation by Rimmmer and Liu, that might reduce the actual optic disc, or the amplification effect of the digital image in vivo.

In our study, we analyzed that birth weight and gestational age had no statistical significance on optic disc parameters at the gestational age of 31 to 40 weeks, which might indicate that the optic disc has developed to full-term birth levels around 31 weeks, which was related to JW Park [24]. In this study, the longitudinal cup-to-disk ratio and transverse cup-to-disk ratio of premature and full-term neonates were not statistically significant, with the values of 0.3 to 0.4.

In view of the fact that Hellstrom et al. pointed out that during childhood (at the age of 7 years) [16], the area of the edge of the optic disc was born 27 weeks before the child was significantly smaller than the full moon. This might due to premature infants, the degree of retinal maturity was different from normal newborns. In the last month of pregnancy, full-term newborns were in a relatively stable environment in the mother’s womb. However, premature infants were in different environments at the corresponding time, and might therefore change their physiology and metabolism. This change would lead to the optic nerve to increase to the maximum diameter at the age of 2 years, interfered with the natural apoptosis of the optic axon leaded to excessive axonal apoptosis [25]. Therefore, the lower weight of premature babies, the lower the gestational age, the larger the cup and the smaller the area along the disc they would have after 2 years old.

In addition, our study observed that the scleral ring morphology of the optic disc has four forms, including acyclic, single ring, double ring, and mixed. Among the 60 eyes of premature infants, 8 were acyclic (10.0 %), 22 were single ring (36.7 %), 17 were double ring (28.3 %), and 15 were mixed (25.0 %). In the full-term newborns, the scleral ring morphology of the optic disc was acyclic (46.7 %), single ring (18.3 %), double ring (6.7 %), and mixed (28.3 %). The data showed that the ratio of the scleral single ring and double ring in the optic disc was significantly higher in the preterm infants than in the full-term newborns, while the acyclic ring ratio of the optic disc in the full-term newborns, significantly more than the proportion of premature infants. Among them, the double ring sign was considered to be a characteristic manifestation of optic nerve hypoplasia [26, 27]. Optic nerve hypoplasia was regarded as abnormal development of the optic nerve, characterized by a decrease in the number of optic nerve fibers in the optic nerve [28] and a small disc morphology [26], which might be only 1/3 − 1/2 of normal, and might be small or nearly normal. Compared the proportion of the scleral double ring and single ring in the optic disc in premature infants and full-term newborns, we believed that the optic disc scleral double ring and single ring was a relatively early naive stage in the development of the optic disc during neonatal development.

Objects in our study ranged from premature infants to full-term newborns, from 31.74 weeks to 40.85 weeks of gestational age, and the proportion of double-rings decreased from 28.3–6.7 % with increasing gestational age. This suggested that due to leaving the relatively stable uterus environment early in the final stages of pregnancy, oxygenation of the placenta changed to pulmonary oxygenation, fetal circulation of premature infants also changed. Oxygen saturation rose from mixed venous blood levels to arterial blood levels, while fetal lungs were still immature and do not perform the oxygen transport process well. These altered functions and metabolic requirements might have certain effects on the morphological development of the optic disc, slowed down the process of maturation of the scleral ring of the optic disc. Therefore, further follow-up studies were necessary for postnatal neonatal optic disc development.

In recent years, development of digital imaging technology, especially the neonatal digital wide-area fundus imaging system made it possible to conduct screening for neonatal eye diseases in ophthalmic hospitals [29, 30]. Neonatal digital wide-area fundus imaging systems overcame some of the shortcomings of traditional inspection equipment. Retcam took only few minutes and could reduce the impact of crying on newborns, especially those with poor general conditions. Screened and recorded of fundus diseases could be completed with a minimum of 5 photos for the entire examination (posterior pole and 4 quadrants). However,Retcam also had limitations: (1) The lens design was more suitable for smaller infants than infants with larger months. (2) Preparation for dilation before the examination of neonatal fundus diseases would cause the time consuming. (3) Children with poor fit need fundus examination under general anesthesia. The cumbersome procedure and invasive anesthesia examination limited the fundus examination of older children.

## Conclusions

In conclusion, our study showed that neonatal digital wide-area fundus imaging system (Retcam3) could not only provide clear fundus imaging images, but also data measurement and analysis of optic disc parameters. The morphology of the optic discs in premature infants and full-term newborns observed in this study was longitudinally elliptical. There was no statistically significant difference between the longitudinal diameter of the optic disc, the transverse diameter of the optic disc, the longitudinal diameter of the optic cup, and the transverse diameter of the optic cup. The use of instrumental tests in prenatal diagnostics and neonatal Retcam should be taken into consideration, to enhance therapies, useful to prevent and treat damage to the optic nerve, secondary to dystocia or premature birth [31].

The types of scleral rings of premature and full-term neonatal optic discs could be divided into four types: acyclic, single-ring, double-ring, and mixed. Among them, the acyclic form represents the mature type of the optic disc scleral ring; the single-ring, the double-ring and the mixed type represent the naive type of the optic disc scleral ring. In premature infants, the naive type of optic disc scleral ring type (single-ring and double-ring) was higher than in term infants. The mature type of optic disc sclera (acyclic) was higher in term newborns than in preterm infants. The optic disc scleral ring mixed type did not differ between preterm and term newborns. The scleral ring double loop sign of the optic disc was a normal stage of optic disc development. The use of Retcam in clinical diagnostics could help give new perspectives on the topic in terms of impact on current practice.

## Data Availability

The datasets used and analyzed during the current study are available from.
